# YKL40 in sporadic amyotrophic lateral sclerosis: cerebrospinal fluid levels as a prognosis marker of disease progression

**DOI:** 10.18632/aging.101551

**Published:** 2018-09-13

**Authors:** Pol Andrés-Benito, Raúl Domínguez, Maria J. Colomina, Franc Llorens, Mònica Povedano, Isidre Ferrer

**Affiliations:** 1 Department of Pathology and Experimental Therapeutics, University of Barcelona, L’Hospitalet de Llobregat, Barcelona, Spain; 2 Biomedical Network Research Center on Neurodegenerative Diseases (CIBERNED), Institute Carlos III, L’Hospitalet de Llobregat, Barcelona, Spain; 3 Bellvitge Biomedical Research Institute (IDIBELL), L’Hospitalet de Llobregat, Barcelona, Spain; 4 Functional Unit of Amyotrophic Lateral Sclerosis (UFELA), Service of Neurology, Bellvitge University Hospital, L’Hospitalet de Llobregat, Barcelona, Spain; 5 Anesthesia and Critical Care Department, Bellvitge University Hospital - University of Barcelona L’Hospitalet de Llobregat, Barcelona, Spain; 6 Neuropathology, Pathologic Anatomy Service, Bellvitge University Hospital, IDIBELL, L’Hospitalet de Llobregat, Barcelona, Spain; 7 Institute of Neurosciences, University of Barcelona, Barcelona, Spain

**Keywords:** amyotrophic lateral sclerosis, chitinase-3-like protein 1, YKL40, spinal cord, frontal cortex area 8, NF-L, cerebrospinal fluid

## Abstract

Amyotrophic lateral sclerosis (ALS) has variable clinical course and fatal outcome. Since inflammation plays a role in the pathogenesis of ALS, chitinase-3-like protein 1 or YKL40 has been assessed as putative biomarker of disease progression. YKL40 mRNA levels are increased in anterior horn of the spinal cord (*P=*0.004) in sporadic ALS (sALS) cases when compared with age-matched controls. These correlate with increased mRNA expression of microglial markers *AIF1* and *CD68* in the spinal cord in sALS (*P=*0.044 and *P=*0.000, respectively). YKL40 mRNA and protein expression had a tendency to increase in post-mortem frontal cortex area 8 (*P=*0.06 and *P=*0.08, respectively). Yet YKL40 immunoreactivity is restricted to a subpopulation of astrocytes in these regions. YKL40 protein levels, as revealed by enzyme-linked immunosorbent assay (ELISA), are significantly increased in the CSF in sALS (n=86) compared with age-matched controls (n=21) (*P*=0.045). Higher levels are found in patients with fast progression when compared with patients with slow and normal progression (*P*=0.008 and *P*=0.004, respectively), and correlates with ALS-FRS-R slope (*P*=0.000). Additionally, increased protein levels of neurofilament light chain (NF-L) are also found in sALS (*P*=0.000); highest values are found in patients with fast progression when compared with cases with slow and normal progression (*P*=0.005 and *P*=0.000, respectively), and also correlate with ALS-FRS-R slope (*P*=0.000). Pearson’s correlation test linked positively the increased levels of YKL40 with increased NF-L levels (*P*=0.013). These data point to YKL40 and NF-L protein levels in the CSF as a good biomarker combination of disease progression in sALS.

## Introduction

Chitinase-3-like protein 1 (CHI3L1) or YKL40 is a glycoprotein with a molecular weight of about 40 kDa that belongs to the family of chitinase-like proteins. Chitinases break down glycosidic bonds in chitin, a component of the cell wall of fungi and the exoskeleton of arthropods [1–5]. YKL40 and other chitinases are also localized in various tissues in vertebrates but their function is not known; YKL40 shows no chitinase activity. Increased expression levels of certain chitinases, and particularly of YKL40, are linked to inflammation, injury, tissue remodelling and regeneration, angiogenesis, and abnormal cell proliferation in tumours [6,7]. Focusing on neurologic diseases, increased YKL40 expression levels have been observed in encephalitis, stroke, traumatic brain injury, multiple sclerosis, and glioblastomas [8–17]. YKL40 expression is also increased in the cerebrospinal fluid (CSF) in neurodegenerative diseases such as Alzheimer’s disease, frontotemporal dementia, and Creutzfeldt-Jakob disease, but not in Parkinson disease or dementia with Lewy bodies [18–36]. For this reason, determination of YKL40 in the CSF has been postulated as a new biomarker that may guide diagnosis in particular clinical settings. Since YKL40 is mainly expressed in astrocytes with only minor expression, if any, in microglia, increased YKL40 in CSF is interpreted as a reactive response of astrocytes linked to inflammation and regeneration [33,37–40].

Chitinases have also been assessed in the brain and biological fluids in amyotrophic lateral sclerosis (ALS). Chitotriosidase (CHIT1) activity is increased in blood in ALS cases when compared with controls, and CHIT1 levels are higher in patients with rapid progression [41]. Furthermore, CHIT1 is increased in microglia and macrophages in spinal cord in ALS, and CSF levels correlate with disease severity and progression [42]. YKL40 and chitinase-3-like protein 2 (CHI3L2) mRNA levels are increased in the motor cortex in ALS [43]. Finally, as determined with liquid chromatography/tandem mass spectrometry, elevated CHIT1, YKL40, and CHI3L2 levels in the CSF correlate with disease progression in ALS [44]. A parallel work presented by another group at the ALS Society Meeting (Amsterdam June 7-8, 2018) reported increased YKL40 in the CSF along the ALS-FTD spectrum [45].

The present study examines YKL40 mRNA and protein expression in brain, YKL40 mRNA levels in blood, and protein levels in the CSF in cases of sporadic ALS (sALS) to learn about the relation between mRNA and protein levels in the central nervous system, and those in CSF and peripheral blood. Based on the previous observations in several diseases, it is worth to have in mind that that YKL40 is not looked as a putative specific biomarker of ALS but as a potential biomarker of prognosis. Therefore, the present study was geared to learn about the use of YKL40 as a possible biomarker of progression in this disease.

## RESULTS

### Increased *CHI3L1* mRNA expression levels in the anterior horn of the spinal cord and frontal cortex in sALS

**Figure 1:**
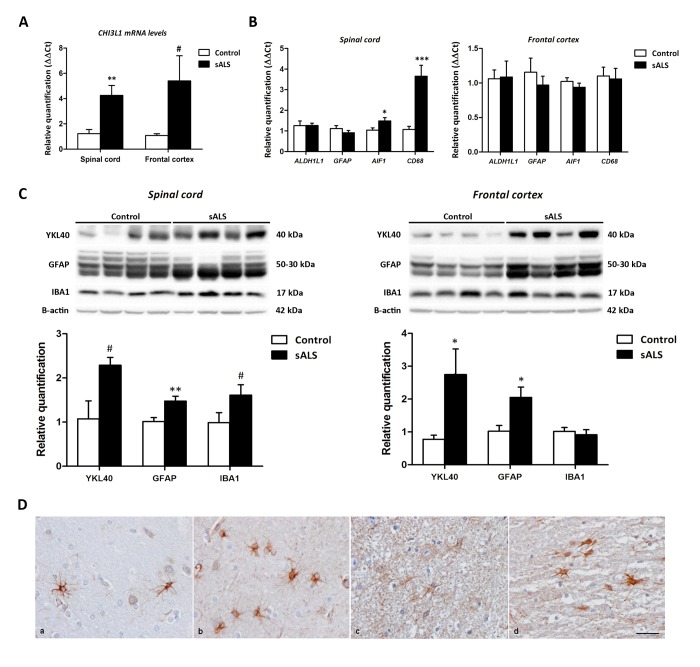
(**A**) *CHI3L1* mRNA expression levels in the anterior horn of the lumbar spinal cord and frontal cortex area 8 in sALS and control cases. *CHI3L1* is significantly up-regulated in the anterior spinal cord but has only a tendency to increase without significance in the frontal cortex in sALS compared with controls. (**B**) mRNA expression levels of microglial (*CD68* and *AIF1*) and astroglial (*GFAP* and *ALDH1L1*) markers in the anterior horn of lumbar spinal cord and frontal cortex area 8 in sALS and age-matched controls. Microglial markers CD68 and AIF1 are significantly up-regulated in the anterior horn of the spinal cord but not in the frontal cortex in sALS. The mRNA expression levels of astroglial markers in the spinal cord and frontal cortex are not modified in pathological cases when compared with controls. (**C**) Western blot analysis of YKL40 in the spinal cord (left panel) and frontal cortex area 8 (right panel) of control and sALS; β-actin was used for normalization. Graphical representation of western blot data; fold changes in the expression of protein are determined relative to the control cases. YKL40 and GFAP protein levels are increased in the spinal cord and frontal cortex in sALS when compared with controls. Due to individual variation, increased values in the anterior horn of the spinal cord showed only a tendency without statistical significance. In contrast, expression levels were not significantly modified in sALS. *P < 0.05, **P < 0.01, and ***P < 0.001, tendency #*P*<0.1. (**D**) YKL40 expression in frontal cortex area 8 (a, b) and spinal cord (c, d) in control (a, c) and sALS (b, d) cases) is found in astrocytes; immunohistochemical sections lightly counterstained with haematoxylin, bar = 25μm.

Significantly increased expression of *CHI3L1* was found in the anterior horn of the spinal cord (*P=*0.004) in sALS (Figure 1A). Levels of transcripts coding for the main markers of astrocytes and microglia were also assessed in the anterior horn of the spinal cord. Significantly up-regulated levels of microglial markers *AIF1* and *CD68* were detected in the spinal cord in sALS (*P=*0.044 and *P=*0.000, respectively) (Figure 1B), which significantly correlated with *CHI3L1* mRNA expression (*P=*0.043 and *P=*0.025, respectively). However, *GFAP* and *ALDH1L1* mRNA levels did not show differences between sALS and control cases (*P*=0.22 and *P*=0.77, respectively) (Figure 1B). No correlations were detected with *CHI3L1* expression (*P=*0.66 and *P*=0.88, respectively) in the spinal cord region.

*CHI3L1* mRNA expression had a tendency to increase in the frontal cortex area 8 in sALS (*P=*0.06) (Figure 1A). No changes were observed in the mRNA levels of *AIF1* (*P*=0.32), *CD68* (*P*=0.89), *GFAP* (*P*=0.15), and *ALDH1L1* (*P*=0.15) in sALS (Figure 1B). Finally, no correlations were found between *CHI3L1* mRNA levels, and astrocytic and microglial markers in frontal cortex area 8 of sALS cases.

### Protein levels of YKL40 are increased in frontal cortex area 8 in sALS

Western blotting showed a tendency to increase YKL40 and IBA1 protein levels in the anterior horn of the spinal cord of sALS when compared with controls (*P*=0.08 and *P*=0.07, respectively). GFAP protein levels were significantly increased, particularly breakdown products (BDP) (*P=*0.01) in the spinal cord of sALS when compared with controls (Figure 1C). Increased GFAP low molecular weight bands (BDPs) have been previously reported in ALS [46]. In contrast, a significant increase in YKL40 (*P=*0.03) and GFAP (*P=*0.02) levels, but not in IBA1 (*P=*0.62), was found in frontal cortex area 8 in sALS when compared with controls (Figure 1C). YKL40 immunoreactivity was restricted to astrocytes in the frontal cortex and spinal cord in sALS and control cases (Figure 1D)

### Levels of YKL40 are increased in CSF of sALS patients and correlate with ALS-FRS evolution and fast disease progression

**Figure 2:**
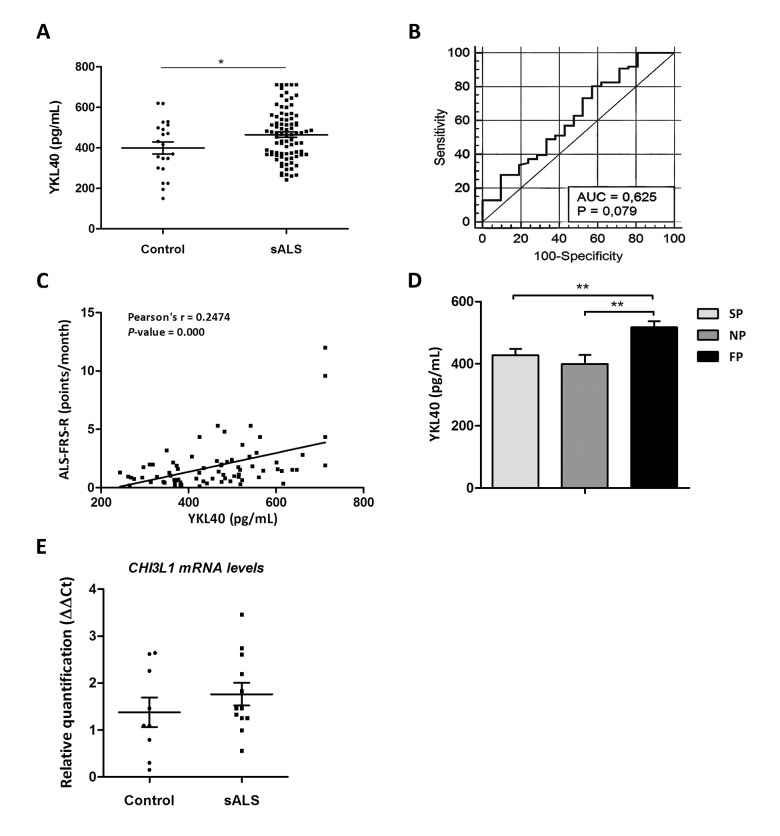
(**A**) Quantification of YKL40 protein levels in the CSF in sALS (n=85) and control (n=23) cases. (**B**) ROC curves for YKL-40 quantification in the differential diagnosis of sALS compared to control cases. In the legend, AUC values, corresponding to the area under ROC curves, and 95% confidence intervals are reported. (**C**) Positive correlation between ALS-FRS-R slope (point/month) and YKL40 levels (pg/mL) (Pearson’s correlation, *P*=0.000). (**D**) Higher YKL40 protein levels in the CSF are found in cases with short survival (fast progression: FP) when compared with cases with slow and normal progression (SP and NP, respectively; *P* = 0.008, *P* = 0.004). (**E**) *CHI3L1* mRNA expression levels in whole-blood samples of sALS and control cases. *CHI3L1* is not deregulated in sALS.

Significantly higher YKL40 levels were detected in sALS cases (465.41 ± 13.45 pg/mL) compared with controls (399 ± 29.52 pg/mL) (*P*=0.045) (Figure 2A). To calculate the clinical accuracy of YKL-40 in discriminating between sALS and the control group, we estimated the AUC value (AUC: 0.6254, 95% CI: 0.52–0.72) (Figure 2B). Considering the optimal cut-off at 356.24pg/mL, defined by the Youden index, an overall sensitivity of 80% and specificity of 43% can be predicted. To demonstrate possible relations between increased levels of YKL40 and the main clinical parameters, Pearson's correlation or parametric comparisons tests were applied. Clinical parameters such as age, gender, disease onset, disease progression, signs of frontotemporal lobar degeneration, and ALS-FRS-R score were examined. Pearson’s correlation indicated a significant link between age and YKL40 levels, increasing with age in controls and sALS (*P*=0.000). Additionally, Pearson’s correlation test demonstrated a positive correlation between ALS-FRS-R slope and YKL40 levels in CSF (*P*=0.004) (Figure 2C). Based on these observations, YKL40 levels were studied in function of the disease progression in every patient; a significant increase in YKL40 CSF levels was identified in those patients with fast progression when compared with patients with slow and normal progression (*P*=0.008 and *P*=0.004, respectively) (Figure 2D). Since at the end of the study only 28 of the 86 sALS cases assessed had died, no attempt was made to analyze the relationship between YKL40 levels in the CSF with survival.

### *CHI3L1* mRNA levels in blood

Additionally, *CHI3L1* mRNA levels were analyzed in whole peripheral blood samples of sALS at the time of diagnosis. Despite the relatively small number of control and disease cases, individual variations were frequent in the two groups and accounted for the lack of significant changes between control and sALS cases (Figure 2E).

### Levels of NF-L are increased in CSF of sALS patients and correlate with ALS-FRS-R slope evolution, fast disease progression and YKL40 levels

**Figure 3:**
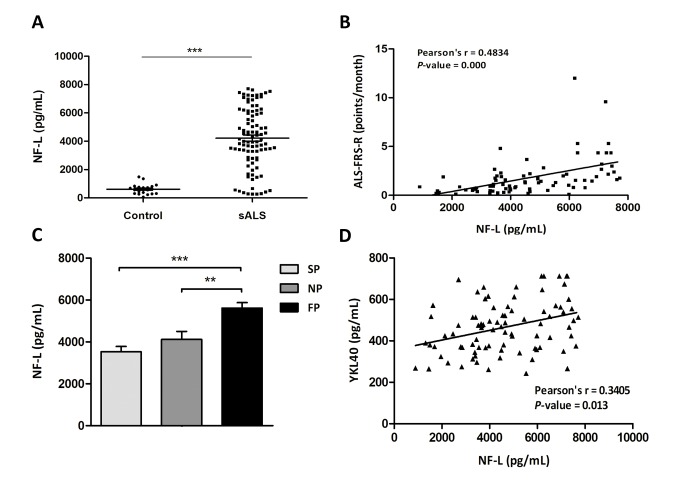
(**A**) Quantification of NF-L protein levels in the CSF in sALS (n=85) and control (n=23) cases. (**B**) Positive correlation between ALS-FRS-R slope (point/month) and NF-L levels (pg/mL) (Pearson’s correlation, *P*=0.000). (**C**) Higher NF-L protein levels in the CSF are found in cases with fast progression (FP) when compared with cases with slow and normal progression (SP and NP, respectively; *P* = 0.000, *P* = 0.005). (**D**) Positive correlation between YKL40 levels (pg/mL) and NF-L levels (pg/mL) (Pearson’s correlation, *P*=0.013).

Neurofilament light chain (NF-L) levels were quantified in CSF of the same cohort of control and sALS cases. Significant higher NF-L levels were detected in sALS cases (4637.55 ± 192.31pg/mL) compared with controls (610.36 ± 81.11pg/mL) (*P*=0.000) (Figure 3A). Additionally, NF-L levels were correlated with ALS-FRS-R slope using Pearson’s test; positive correlation was found between ALS-FRS-R and NF-L levels in CSF (*P*=0.000) (Figure 2B). NF-L levels were significantly increased in patients with fast progression when compared with patients with slow and normal progression (*P*=0.000 and *P*=0.005, respectively) (Figure 2C). Finally, positive significant correlation was observed between YKL40 and NF-L levels (Pearson’s correlation test, *P*=0.013) (Figure 2D).

## DISCUSSION

Inflammation involving microglial cells, macrophages, T cells, astrocytes, and neurons, and mediated by a plethora of mediators of the immune response including Toll-like receptors, members of the complement system, pro- and anti-inflammatory cytokines, chemoquines, and blood vessel factors, are activated in the anterior horn of the spinal cord and, to a lesser extent, in other brain regions in ALS [47–59].

Increased protein levels of several cytokines and mediators of the inflammatory response have also been reported in the CSF in ALS, including IL-1, IL-1β, IL-6, IL-8, IL-12, IL-15, IL-17A, IL-18BP, IL-23, RANTES, chemokines, and MCP1 [60–69]. This heterogeneous representation indicates variations depending on the methods and products employed in the different laboratories. Moreover, CSF profiles of angiogenic and inflammatory factors are, at least in part, dependent on the respiratory status of ALS patients [70].

Increased levels of selected inflammatory markers are found in blood and serum in ALS, thus suggesting systemic inflammatory responses which roughly correlate with disease progression [71–80].

All these observations strongly support a role of inflammation in the pathogenesis of sALS. However, the identification of a biomarker of inflammation with practical prognosis value has been limited because of individual variation and variations between methods and laboratories.

Previous studies in ALS have shown YKL40 mRNA up-regulation in the motor cortex [43] and increased YKL40 protein levels in the CSF correlating with disease progression [44,45]. Regarding brain tissue, the present observations show significant YKL40 mRNA up-regulation in the anterior horn of the spinal cord and frontal cortex area 8, accompanied by significantly increased YKL40 protein levels in the frontal cortex and a tendency to increased YKL40 in the spinal cord in sALS. Importantly, YKL40 is expressed in astrocytes, in agreement with other observations [33,36–40], but in contrast to another description ascribing YKL40 expression to brain macrophages [44].

Up-regulation and increased YKL40 expression occcurs in parallel with increased values of microglia markers in the spinal cord but not in the frontal cortex area in sALS, and with increased GFAP protein levels in the spinal cord and frontal cortex but not with GFAP mRNA up-regulation in these regions.

Together, these observations point to earlier responses in astrocytes when compared with microglial reactions in the frontal cortex in sALS, whereas microglial markers are strongly expressed in the spinal cord in the same group of patients.

Based on these findings, increased YKL40 protein levels in the CSF mirror YKL40 changes in the central nervous system, and they can be interpreted as the consequence of YKL40 delivery of astrocytes to the CSF. Unfortunately, no analysis of a possible correlation between YKL40 brain and spinal cord values, and disease progression/survival, was feasible in the present series because of the lack of sufficient clinical data. However, YKL40 CSF values negatively correlate with patient survival, thus indicating that higher YKL40 in the CSF likely occurs in patients with rapid disease progression.

We do not know at this time what the functional implications of elevated YKL40 expression in ALS and other neurological diseases are. Nor do we know whether YKL40, even considering this particular chitinase as a marker of astrocyte inflammation, has beneficial or deleterious effects. In this line, *chi3l1* KO mice have increased astrocytic responses (GFAP staining) and increased IBA1 microglial expression when compared with wild-type animals following traumatic brain injury, thus suggesting that YKL40 limits the extent of astroglial and microglial neuroinflammation [38]. If this is the case then increased YKL40 expression *per se* would not be dangerous but rather a manifestation of increased beneficial response in the face of a more aggressive facet of ALS in a subgroup of patients.

The present findings point to the likelihood that increased YKL40 levels in the CSF are not disease specific but they are good biomarker of disease progression in sALS.

Previous studies have shown increased levels of neurofilaments in the CSF of ALS cases [81–84]. NF heavy chain levels in CSF were negatively correlated disease duration and ALS-FRS-R slope, and NF-L light chain levels in CSF were negatively correlated with disease duration. Thus, NF heavy and light chain levels have potential use as markers of neural degeneration in ALS [85,86]. Increased NF-L in the CSF are not either specific for the disease, but they are more likely used as measures of disease progression [85,86].

In the present work, YKL40 levels in the CSF were assessed in parallel with levels of NF light chain. As expected, our results are in line with previous observations by other authors; NF-light chain levels are significantly increased in ALS and levels negatively correlate with disease progression and ALS-FRS-R slope in our series.

In summary, the present findings point that YKL40 and NF-L levels in CSF constitute valuable combination of biomarkers for improving accuracy in the prognosis of patients with sALS.

## MATERIALS AND METHODS

### Tissue samples

Post-mortem fresh-frozen lumbar spinal cord (SC) and frontal cortex (FC) (Brodmann area 8) tissue samples were obtained from the Institute of Neuropathology HUB-ICO-IDIBELL Biobank following the guidelines of Spanish legislation on this matter and the approval of the local ethics committee. The post-mortem interval between death and tissue processing was between 2h and 17h. One hemisphere was immediately cut in coronal sections, 1-cm thick, and selected areas of the encephalon were rapidly dissected, frozen on metal plates over dry ice, placed in individual air-tight plastic bags, numbered with water-resistant ink, and stored at -80°C until use for biochemical studies. The other hemisphere was fixed by immersion in 4% buffered formalin for 3 weeks for morphologic studies. Transversal sections of the spinal cord were alternatively frozen at -80°C or fixed by immersion in 4% buffered formalin. The anterior horn of the lumbar spinal cord was dissected on a dry-ice frozen plate under a binocular microscope at a magnification x4.

The neuropathological study was carried out on paraffin sections of twenty-six selected regions of the cerebrum, cerebellum, brain stem, and spinal cord which were stained with haematoxylin and eosin, Klüver Barrera, periodic acid shiff, and processed for immunohistochemistry with anti-β-amyloid, phospho-tau (clone AT8), α-synuclein, αB-crystallin, TDP-43, ubiquitin, p62, glial fibrillary acidic protein, CD68, and IBA1 [87]. All cases met the neuropathological criteria for classical ALS regarding involvement of motor cortex, pyramidal tracts, and selected motor nuclei of the cranial nerves and anterior horn of the spinal cord [88,89]. In addition, TDP-43-immunoreactive small dystrophic neurites and TDP-43-positive cytoplasmic neuronal inclusions in frontal cortex area 8 were observed in 11 of 18 cases, but they were abundant only in three cases (cases 29, 30, and 31). Spongiosis in the upper cortical layers was found in only one case (case 28). Frontotemporal dementia was found in no cases of the present series.

Patients with associated pathology including Alzheimer disease (excepting neurofibrillary tangle, NFT, pathology stages I-II of Braak and Braak), Parkinson disease, tauopathies, vascular diseases, neoplastic diseases affecting the nervous system, metabolic syndrome, hypoxia, and prolonged axonal states such as those occurring in intensive care units were excluded. Cases with infectious, inflammatory, or autoimmune diseases, either systemic or limited to the nervous system, were not included. Age-matched control cases had not suffered from neurologic or psychiatric diseases, and did not have abnormalities in the neuropathological examination excepting NFT pathology stages I-II of Braak and Braak. A summary of sALS and control cases is shown in Table 1.

**Table 1:** Summary of cases with tissue samples.

						**RIN value**	
**Case**	**Age**	**Gender**	**Diagnosis**	**PM delay**	**Initial symptoms**	**SC**	**FC**
1	49	F	Control	07 h 00 min	-	-	7.2
2	75	F	Control	03 h 00 min	-	-	7.2
3	55	M	Control	05 h 40 min	-	-	7.7
4	59	M	Control	12 h 05 min	-	6.4	-
5	59	M	Control	07 h 05 min	-	-	7.8
6	43	M	Control	05 h 55 min	-	6.6	7.7
7	53	M	Control	07 h 25 min	-	-	5.3
8	56	M	Control	03 h 50 min	-	-	7.6
9	47	M	Control	04 h 55 min	-	5.6	7.7
10	64	F	Control	11 h 20 min	-	6.2	-
11	46	M	Control	15 h 00 min	-	5.9	7.9
12	56	M	Control	07 h 10 min	-	6.1	-
13	71	F	Control	08 h 30 min	-	5.9	-
14	64	F	Control	05 h 00 min	-	7.0	-
15	79	F	Control	06 h 25 min	-	6.7	-
16	75	M	Control	07 h 30 min	-	5.0	-
17	55	M	Control	09 h 45 min	-	5.3	-
18	52	M	Control	03 h 00 min	-	-	8.3
19	52	M	Control	04 h 40 min	-	-	6.3
20	76	M	Control	06 h 30 min	-	6.6	-
21	60	F	Control	11 h 30 min	-	-	7.5
22	51	F	Control	04 h 00 min	-	6.3	7.9
23	54	M	Control	08 h 45 min	-	-	7.0
24	56	M	ALS	10 h 50 min	NA	7.1	-
25	70	M	ALS	03 h 00 min	Respiratory	7.3	7.0
26	77	M	ALS	04 h 30 min	NA	7.4	-
27	56	F	ALS	03 h 45 min	NA	8.2	7.7
28	59	M	ALS	03 h 15 min	NA	7.5	7.7
29	63	F	ALS	13 h 50 min	Bulbar	6.8	8.2
30	59	F	ALS	14 h 15 min	NA	6.4	6.7
31	54	M	ALS	04 h 50 min	Spinal	-	7.8
32	76	M	ALS	12 h 40 min	Spinal	-	7.4
33	64	M	ALS	16 h 30 min	NA	6.3	7.3
34	57	F	ALS	04 h 00 min	Bulbar	6.2	8.6
35	75	F	ALS	04 h 05 min	Bulbar	6.8	6.8
36	79	F	ALS	02 h 10 min	NA	7.0	-
37	57	F	ALS	10 h 00 min	Bulbar	6.5	7.1
38	50	M	ALS	10 h 10 min	Spinal	-	5.9
39	59	F	ALS	02 h 30 min	Spinal	-	7.5
40	46	M	ALS	07 h 00 min	Spinal	7.0	8.0
41	69	F	ALS	17 h 00 min	Spinal	6.4	6.3

Abbreviations: F: female; M: male; PM delay: post-mortem delay; SC: spinal cord; FC: frontal cortex; RIN: RNA integrity number.

### CSF collection

Cerebrospinal fluid (CSF) was collected prospectively from patients undergoing lumbar puncture due to clinical suspicion of motor neuron disease at the functional unit of amyotrophic lateral sclerosis (UFELA) of the Neurology Service of the Bellvitge University Hospital. Samples were obtained from 86 sALS patients (Table 2). In these patients, 1.5 ± 0.5mL of CSF was collected in polypropylene tubes as part of the clinical routine investigation. CSF was centrifuged at 3,000 rpm for 15 min at room temperature. Supernatant was collected and aliquoted in volumes of 250μL and stored at -80ºC until use. All samples were analyzed after one freeze/thaw cycle.

**Table 2:** Summary of cases with CSF samples.

**Group**	**n**	**Gender**	**Initial symptoms**	
Control	21	9 (M) + 12 (F)	-	
	86	47 (M) + 39 (F)	Spinal	55
sALS			Bulbar	29
			Respiratory	2

Abbreviations: F: female; M: male; NA: not available.

Patients were evaluated clinically according to the main signs at onset (spinal, bulbar, and respiratory) and categorized according to disease progression as fast, expected, and slow progression depending on the survival or the clinical evolution in those still alive. Fast progression was considered in patients who survived less than 3 years; normal progression was considered between 3 and 5 years, and slow progression for those still alive after 5 years. The ALS Functional Rating Scale Revised (ALS-FRS-R, version May 2015) was used in every case. CSF from control cases was obtained from 21 healthy donors following the protocols for the use of biological samples for research (Table 2). No ALS cases or controls suffered from infection or inflammatory disorder at the time of sampling. CSF samples from sALS cases and age-matched controls were obtained after signed informed consent and approval by the Clinical Research Ethics Committee (CEIC) of the Bellvitge University Hospital.

### Whole blood collection

Whole blood samples were collected using PAXgene Blood RNA Tube (PAXgene Blood RNA Tube, PreAnalytiX, Qiagen® GmbH, Hilden, GE) collecting system. Two PAXgene Blood RNA tubes were obtained per case. Samples were collected at the first visit once the clinical diagnosis was established (n=12 sALS, n=10 controls).Tubes were kept for 2 h at room temperature to ensure lysis of blood cells, and then stored at −20°C for 24 h. Thereafter, tubes were stored at −80°C for at least 7 days prior to processing. A summary of sALS and control cases is shown in Table 3.

**Table 3:** Summary of cases for whole peripheral blood mRNA studies.

**Case**	**Age**	**Gender**	**Diagnosis**	**Initial symptoms**	**RIN value**
1	60	M	Control	-	9.1
2	68	M	Control	-	9.2
3	66	F	Control	-	9.0
4	N/A	M	Control	-	8.9
5	74	M	Control	-	8.0
6	N/A	F	Control	-	8.3
7	67	M	Control	-	6.1
8	72	F	Control	-	6.0
9	44	F	Control	-	6.0
10	66	F	Control	-	6.1
11	60	M	ALS	Spinal	7.4
12	63	M	ALS	Spinal	8.7
13	66	F	ALS	Bulbar	8.9
14	53	F	ALS	Bulbar	7.3
15	73	M	ALS	Bulbar	8.6
16	65	M	ALS	Spinal	8.9
17	43	M	ALS	Bulbar	8.6
18	57	F	ALS	Bulbar	7.4
19	65	M	ALS	Bulbar	7.1
20	67	M	ALS	Bulbar	7.4
21	73	M	ALS	Spinal	6.1
22	73	F	ALS	Spinal	6.0

Abbreviations: F: female; M: male; NA: not available; RIN: RNA integrity value.

### Genetic studies

Genetic testing was performed on genomic DNA isolated from blood or brain tissue. Informed consent for the chromosome9 open reading frame (*C9ORF72*), superoxide dismutase 1 (*SOD1*), TAR DNA binding protein (*TARDBP*), and FUS RNA binding protein (*FUS*) analysis was obtained from each patient or legal representative. Patients in this study did not show mutations in the assessed genes.

### RNA extraction and RT-qPCR

RNA from dissected frozen anterior horn of the lumbar spinal cord (n=14 sALS, n=13 controls) and frontal cortex area 8 (n=15 sALS, n=14 controls) was extracted following the instructions of the supplier (RNeasy Mini Kit, Qiagen® GmbH, Hilden, Germany). PAXgene Blood RNA tubes were incubated overnight at 4°C in a shaker-plate to equilibrate the temperature and to increase yields, and then at room temperature for 2h before starting the procedure. RNA from frozen whole blood samples was extracted following the instructions of the supplier (PAXgene Blood RNA kit, PreAnalytiX, Qiagen® GmbH, Hilden, GE). RNA integrity and 28S/18S ratios were determined with the Agilent Bioanalyzer (Agilent Technologies Inc, Santa Clara, CA, USA) to assess RNA quality, and the RNA concentration was evaluated using a NanoDrop™ Spectrophotometer (Thermo Fisher Scientific). Complementary DNA (cDNA) preparation used the High-Capacity cDNA Reverse Transcription kit (Applied Biosystems, Foster City, CA, USA) following the protocol provided by the supplier. Parallel reactions for each RNA sample were run in the absence of MultiScribe Reverse Transcriptase to assess the lack of contamination of genomic DNA. TaqMan RT-qPCR assays were performed in duplicate for each gene on cDNA samples in 384-well optical plates using an ABI Prism 7900 Sequence Detection system (Applied Biosystems, Life Technologies, Waltham, MA, USA). For each 10μL TaqMan reaction, 4.5μL cDNA was mixed with 0.5μL 20x TaqMan Gene Expression Assays and 5μL of 2x TaqMan Universal PCR Master Mix (Applied Biosystems). Taqman probes used in expression assays were: allograft inflammatory factor (*AIF1*) (Hs00741549_g1) coding for IBA1, aldehyde dehydrogenase 1 family member L1 (*ALDH1L1*) (Hs01003842_m1), glial fibrillary acidic protein (*GFAP*) (Hs00909233_m1), and *CHI3L1* (Hs01072228_m1). Hypoxanthine-guanine phosphoribosyltransferase (*HPRT1*) was used as internal control for normalization of spinal cord samples, whereas β-glucuronidase (*GUS-β*) was used as the internal control for normalization of frontal cortex samples [90,91]. Mean values of two house-keeping genes, glucuronidase beta (*GUS-β*) [92] and glyceraldehyde 3-phosphate dehydrogenase (*GAPDH*) [93], were used as internal controls for normalization of whole-blood mRNA expression studies.

The parameters of the reactions were 50°C for 2min, 95°C for 10min, and 40 cycles of 95°C for 15sec and 60°C for 1min. Finally, the capture of all TaqMan PCR data used the Sequence Detection Software (SDS version 2.2.2, Applied Biosystems). The double-delta cycle threshold (ΔΔCT) method was utilized to analyze the data results with Student’s-*t* test.

### Gel electrophoresis and immunoblotting

Frozen samples of frontal cortex area 8 (n=6 sALS, n=6 controls) and anterior horn of the spinal cord at the lumbar level (n=4 sALS, n=4 controls) were homogenized in RIPA lysis buffer composed of 50mM Tris/HCl buffer, pH 7.4 containing 2mM EDTA, 0.2% Nonidet P-40, 1mM PMSF, protease and phosphatase inhibitor cocktail (Roche Molecular Systems, USA). The homogenates were centrifuged for 20 min at 12,000 rpm. Protein concentration was determined with the BCA method (Thermo Scientific). Equal amounts of protein (12μg) for each sample were loaded and separated by electrophoresis on 10% sodium dodecyl sulfate polyacrylamide gel electrophoresis (SDS-PAGE) gels and then transferred onto nitrocellulose membranes (Amersham, Freiburg, GE). Non-specific bindings were blocked by incubation in 3% albumin in PBS containing 0.2% Tween for 1h at room temperature. After washing, the membranes were incubated overnight at 4°C with antibodies against glial fibrillary acidic protein (GFAP) (dilution of 1:500; rabbit polyclonal monoclonal, MO761, Dako, Agilent, Santa Clara, USA), ionized calcium binding adapter molecule 1 (IBA1) for microglia (diluted at 1:1,000; rabbit polyclonal, 019-19741, WAKO, Fujifilm, Tokyo, Japan), and YKL-40 (diluted 1:200; goat polyclonal, AF-2599, R&D Systems, Minneapolis, MN, USA). Protein loading was monitored using an antibody against β-actin (42 kDa, 1:30,000, Sigma). Membranes were incubated for 1h with appropriate HRP-conjugated secondary antibodies (1:2,000, Dako); the immunoreaction was revealed with a chemiluminescence reagent (ECL, Amersham). Densitometric quantification was carried out with the ImageLab v4.5.2 software (BioRad), using β-actin for normalization. Six samples per group were analyzed.

### Immunohistochemistry

De-waxed sections, 4μm thick, of the lumbar spinal cord (n=6 sALS, n=6 controls) and frontal cortex area 8 (n=6 sALS, n=6 controls) were processed in parallel for immunohistochemistry. Endogenous peroxidases were blocked by incubation in 10% methanol-1% H_2_O_2_ for 15min followed by 3% normal horse serum. Then the sections were incubated at 4°C overnight with anti-YKL40 primary antibody (PA5-43746, ThermoFisher, Waltham, Massachusetts, USA) at a dilution of 1:200. Immediately afterwards, the sections were incubated with EnVision + system peroxidase (Dako, Agilent, Santa Clara, CA, USA) for 30min at room temperature. The peroxidase reaction was visualized with diaminobenzidine and H_2_O_2_. No signal was obtained following incubation with only the secondary antibody. Sections were slightly stained with haematoxylin.

### Enzyme-linked immunosorbent assays (ELISA) in CSF

YKL40 protein levels were measured using the MicroVue YKL40 EIA ELISA kit (Quidel, San Diego, CA, USA) following the manufacturer’s instructions. Receiver operating characteristic (ROC) curves and derived area under the curve (AUC) were calculated. The best cut-off value, sensitivity, and specificity were estimated based on the Youden index (point on a ROC curve providing the best balance of both sensitivity and specificity) [94]. NF-L levels were measured using the NF-light® (Neurofilament light) ELISA kit from UmanDiagnostics (Umea, Sweden) following the manufacturer’s instructions.

### Statistical analysis

The normality of distribution was analyzed with the Kolmogorov-Smirnov test. The unpaired Student’s *t*-test was used to compare each group when values followed normal distribution, and statistical analysis of the CSF protein data between groups was carried out using one-way analysis of variance (ANOVA) followed by Tukey post-test, in both cases using the SPSS software (IBM Corp. Released 2013, IBM-SPSS Statistics for Windows, Version 21.0., Armonk, NY, USA). Graphic design was performed with GraphPad Prism version 5.01 (La Jolla, CA, USA). Outliers were detected using the GraphPad software QuickCalcs (*p* < 0.05). The data were expressed as mean ± SEM, and significance levels were set at *P < 0.05, **P < 0.01, and ***P < 0.001, and tendencies at #*P* < 0.1. Pearson’s correlation coefficient was used to assess a possible linear association between two continuous quantitative variables.

## References

[1] Adrangi S, Faramarzi MA. From bacteria to human: a journey into the world of chitinases. Biotechnol Adv. 2013; 31:1786–95. 10.1016/j.biotechadv.2013.09.012 24095741

[2] Karthik N, Akanksha K, Pandey A. Production, purification and properties of fungal chitinases--a review. Indian J Exp Biol. 2014; 52:1025–35. 25434097

[3] Rathore AS, Gupta RD. Chitinases from bacteria to human: properties, applications, and future perspectives. Enzyme Res. 2015; 2015:791907. 10.1155/2015/791907 26664744 PMC4668315

[4] Di Rosa M, Distefano G, Zorena K, Malaguarnera L. Chitinases and immunity: ancestral molecules with new functions. Immunobiology. 2016; 221:399–411. 10.1016/j.imbio.2015.11.014 26686909

[5] Langner T, Göhre V. Fungal chitinases: function, regulation, and potential roles in plant/pathogen interactions. Curr Genet. 2016; 62:243–54. 10.1007/s00294-015-0530-x 26527115

[6] Shao R, Hamel K, Petersen L, Cao QJ, Arenas RB, Bigelow C, Bentley B, Yan W. YKL-40, a secreted glycoprotein, promotes tumor angiogenesis. Oncogene. 2009; 28:4456–68. 10.1038/onc.2009.292 19767768 PMC2795793

[7] Lee CG, Da Silva CA, Dela Cruz CS, Ahangari F, Ma B, Kang MJ, He CH, Takyar S, Elias JA. Role of chitin and chitinase/chitinase-like proteins in inflammation, tissue remodeling, and injury. Annu Rev Physiol. 2011; 73:479–501. 10.1146/annurev-physiol-012110-142250 21054166 PMC3864643

[8] Pelloski CE, Mahajan A, Maor M, Chang EL, Woo S, Gilbert M, Colman H, Yang H, Ledoux A, Blair H, Passe S, Jenkins RB, Aldape KD. YKL-40 expression is associated with poorer response to radiation and shorter overall survival in glioblastoma. Clin Cancer Res. 2005; 11:3326–34. 10.1158/1078-0432.CCR-04-1765 15867231

[9] Bonneh-Barkay D, Bissel SJ, Wang G, Fish KN, Nicholl GC, Darko SW, Medina-Flores R, Murphey-Corb M, Rajakumar PA, Nyaundi J, Mellors JW, Bowser R, Wiley CA. YKL-40, a marker of simian immunodeficiency virus encephalitis, modulates the biological activity of basic fibroblast growth factor. Am J Pathol. 2008; 173:130–43. 10.2353/ajpath.2008.080045 18556781 PMC2438291

[10] Bonneh-Barkay D, Wang G, Starkey A, Hamilton RL, Wiley CA. In vivo CHI3L1 (YKL-40) expression in astrocytes in acute and chronic neurological diseases. J Neuroinflammation. 2010 a; 7:34. 10.1186/1742-2094-7-34 20540736 PMC2892443

[11] Bonneh-Barkay D, Zagadailov P, Zou H, Niyonkuru C, Figley M, Starkey A, Wang G, Bissel SJ, Wiley CA, Wagner AK. YKL-40 expression in traumatic brain injury: an initial analysis. J Neurotrauma. 2010 b; 27:1215–23. 10.1089/neu.2010.1310 20486806 PMC2942903

[12] Horbinski C, Wang G, Wiley CA. YKL-40 is directly produced by tumor cells and is inversely linked to EGFR in glioblastomas. Int J Clin Exp Pathol. 2010; 3:226–37. 20224722 PMC2836500

[13] Comabella M, Fernández M, Martin R, Rivera-Vallvé S, Borrás E, Chiva C, Julià E, Rovira A, Cantó E, Alvarez-Cermeño JC, Villar LM, Tintoré M, Montalban X. Cerebrospinal fluid chitinase 3-like 1 levels are associated with conversion to multiple sclerosis. Brain. 2010; 133:1082–93. 10.1093/brain/awq035 20237129

[14] Iwamoto FM, Hormigo A. Unveiling YKL‐40, from serum marker to target therapy in glioblastoma. Front Oncol. 2014; 4:90. 10.3389/fonc.2014.00090 24809021 PMC4009441

[15] Burman J, Raininko R, Blennow K, Zetterberg H, Axelsson M, Malmeström C. YKL-40 is a CSF biomarker of intrathecal inflammation in secondary progressive multiple sclerosis. J Neuroimmunol. 2016; 292:52–57. 10.1016/j.jneuroim.2016.01.013 26943959

[16] Mañé-Martínez MA, Olsson B, Bau L, Matas E, Cobo-Calvo Á, Andreasson U, Blennow K, Romero-Pinel L, Martínez-Yélamos S, Zetterberg H. Glial and neuronal markers in cerebrospinal fluid in different types of multiple sclerosis. J Neuroimmunol. 2016; 299:112–17. 10.1016/j.jneuroim.2016.08.004 27725108

[17] Quintana E, Coll C, Salavedra-Pont J, Muñoz-San Martín M, Robles-Cedeño R, Tomàs-Roig J, Buxó M, Matute-Blanch C, Villar LM, Montalbán X, Comabella M, Perkal H, Gich J, Ramió-Torrentà L. Cognitive impairment in early stages of multiple sclerosis is associated with high cerebrospinal fluid levels of chitinase 3-like 1 and neurofilament light chain. Eur J Neurol. 2018; 25:1189–91. 10.1111/ene.13687 29797629

[18] Craig-Schapiro R, Perrin RJ, Roe CM, Xiong C, Carter D, Cairns NJ, Mintun MA, Peskind ER, Li G, Galasko DR, Clark CM, Quinn JF, D’Angelo G, et al. YKL-40: a novel prognostic fluid biomarker for preclinical Alzheimer’s disease. Biol Psychiatry. 2010; 68:903–12. 10.1016/j.biopsych.2010.08.025 21035623 PMC3011944

[19] Perrin RJ, Craig-Schapiro R, Malone JP, Shah AR, Gilmore P, Davis AE, Roe CM, Peskind ER, Li G, Galasko DR, Clark CM, Quinn JF, Kaye JA, et al. Identification and validation of novel cerebrospinal fluid biomarkers for staging early Alzheimer’s disease. PLoS One. 2011; 6:e16032. 10.1371/journal.pone.0016032 21264269 PMC3020224

[20] Olsson B, Constantinescu R, Holmberg B, Andreasen N, Blennow K, Zetterberg H. The glial marker YKL-40 is decreased in synucleinopathies. Mov Disord. 2013 a; 28:1882–85. 10.1002/mds.25589 23847144

[21] Olsson B, Hertze J, Lautner R, Zetterberg H, Nägga K, Höglund K, Basun H, Annas P, Lannfelt L, Andreasen N, Minthon L, Blennow K, Hansson O. Microglial markers are elevated in the prodromal phase of Alzheimer’s disease and vascular dementia. J Alzheimers Dis. 2013 b; 33:45–53. 10.3233/JAD-2012-120787 22890100

[22] Antonell A, Mansilla A, Rami L, Lladó A, Iranzo A, Olives J, Balasa M, Sánchez-Valle R, Molinuevo JL. Cerebrospinal fluid level of YKL-40 protein in preclinical and prodromal Alzheimer’s disease. J Alzheimers Dis. 2014; 42:901–08. 10.3233/JAD-140624 25024322

[23] Wildsmith KR, Schauer SP, Smith AM, Arnott D, Zhu Y, Haznedar J, Kaur S, Mathews WR, Honigberg LA. Identification of longitudinally dynamic biomarkers in Alzheimer’s disease cerebrospinal fluid by targeted proteomics. Mol Neurodegener. 2014; 9:22. 10.1186/1750-1326-9-22 24902845 PMC4061120

[24] Alcolea D, Carmona-Iragui M, Suárez-Calvet M, Sánchez-Saudinós MB, Sala I, Antón-Aguirre S, Blesa R, Clarimón J, Fortea J, Lleó A. Relationship between β-Secretase, inflammation and core cerebrospinal fluid biomarkers for Alzheimer’s disease. J Alzheimers Dis. 2014; 42:157–67. 10.3233/JAD-140240 24820015

[25] Rosén C, Andersson CH, Andreasson U, Molinuevo JL, Bjerke M, Rami L, Lladó A, Blennow K, Zetterberg H. Increased levels of chitotriosidase and YKL-40 in cerebrospinal fluid from patients with Alzheimer’s disease. Dement Geriatr Cogn Disord Extra. 2014; 4:297–304. 10.1159/000362164 25254036 PMC4164083

[26] Kester MI, Teunissen CE, Sutphen C, Herries EM, Ladenson JH, Xiong C, Scheltens P, van der Flier WM, Morris JC, Holtzman DM, Fagan AM. Cerebrospinal fluid VILIP-1 and YKL-40, candidate biomarkers to diagnose, predict and monitor Alzheimer’s disease in a memory clinic cohort. Alzheimers Res Ther. 2015; 7:59. 10.1186/s13195-015-0142-1 26383836 PMC4574487

[27] Alcolea D, Vilaplana E, Pegueroles J, Montal V, Sánchez-Juan P, González-Suárez A, Pozueta A, Rodríguez-Rodríguez E, Bartrés-Faz D, Vidal-Piñeiro D, González-Ortiz S, Medrano S, Carmona-Iragui M, et al. Relationship between cortical thickness and cerebrospinal fluid YKL-40 in predementia stages of Alzheimer’s disease. Neurobiol Aging. 2015; 36:2018–23. 10.1016/j.neurobiolaging.2015.03.001 25865441

[28] Wennström M, Surova Y, Hall S, Nilsson C, Minthon L, Hansson O, Nielsen HM. The inflammatory marker YKL-40 is elevated in cerebrospinal fluid from patients with Alzheimer’s but not Parkinson’s disease or dementia with Lewy bodies. PLoS One. 2015; 10:e0135458. 10.1371/journal.pone.0135458 26270969 PMC4536228

[29] Gispert JD, Monté GC, Falcon C, Tucholka A, Rojas S, Sánchez-Valle R, Antonell A, Lladó A, Rami L, Molinuevo JL. CSF YKL-40 and pTau181 are related to different cerebral morphometric patterns in early AD. Neurobiol Aging. 2016; 38:47–55. 10.1016/j.neurobiolaging.2015.10.022 26827642

[30] Janelidze S, Hertze J, Zetterberg H, Landqvist Waldö M, Santillo A, Blennow K, Hansson O. Cerebrospinal fluid neurogranin and YKL-40 as biomarkers of Alzheimer’s disease. Ann Clin Transl Neurol. 2015; 3:12–20. 10.1002/acn3.266 26783546 PMC4704480

[31] Teunissen CE, Elias N, Koel-Simmelink MJ, Durieux-Lu S, Malekzadeh A, Pham TV, Piersma SR, Beccari T, Meeter LH, Dopper EG, van Swieten JC, Jimenez CR, Pijnenburg YA. Novel diagnostic cerebrospinal fluid biomarkers for pathologic subtypes of frontotemporal dementia identified by proteomics. Alzheimers Dement (Amst). 2016; 2:86–94. 10.1016/j.dadm.2015.12.004 27239539 PMC4879654

[32] Baldacci F, Toschi N, Lista S, Zetterberg H, Blennow K, Kilimann I, Teipel S, Cavedo E, Dos Santos AM, Epelbaum S, Lamari F, Dubois B, Floris R, et al. Two-level diagnostic classification using cerebrospinal fluid YKL-40 in Alzheimer’s disease. Alzheimers Dement. 2017; 13:993–1003. 10.1016/j.jalz.2017.01.021 28263742

[33] Llorens F, Thüne K, Tahir W, Kanata E, Diaz-Lucena D, Xanthopoulos K, Kovatsi E, Pleschka C, Garcia-Esparcia P, Schmitz M, Ozbay D, Correia S, Correia Â, et al. YKL-40 in the brain and cerebrospinal fluid of neurodegenerative dementias. Mol Neurodegener. 2017; 12:83. 10.1186/s13024-017-0226-4 29126445 PMC5681777

[34] Alcolea D, Vilaplana E, Suárez-Calvet M, Illán-Gala I, Blesa R, Clarimón J, Lladó A, Sánchez-Valle R, Molinuevo JL, García-Ribas G, Compta Y, Martí MJ, Piñol-Ripoll G, et al. CSF sAPPβ, YKL-40, and neurofilament light in frontotemporal lobar degeneration. Neurology. 2017; 89:178–88. 10.1212/WNL.0000000000004088 28592456

[35] Vijverberg EG, Schouws S, Meesters PD, Verwijk E, Comijs H, Koene T, Schreuder C, Beekman A, Scheltens P, Stek M, Pijnenburg Y, Dols A. Cognitive deficits in patients with neuropsychiatric symptoms: A comparative study between behavioral variant frontotemporal dementia and primary psychiatric disorders. J Clin Psychiatry. 2017; 78:e940–46. 10.4088/JCP.16m11019 28749089

[36] Bonneh-Barkay D, Zagadailov P, Zou H, Niyonkuru C, Figley M, Starkey A, Wang G, Bissel SJ, Wiley CA, Wagner AK. YKL-40 expression in traumatic brain injury: an initial analysis. J Neurotrauma. 2010; 27:1215–23. 10.1089/neu.2010.1310 20486806 PMC2942903

[37] Bonneh-Barkay D, Bissel SJ, Kofler J, Starkey A, Wang G, Wiley CA. Astrocyte and macrophage regulation of YKL-40 expression and cellular response in neuroinflammation. Brain Pathol. 2012; 22:530–46. 10.1111/j.1750-3639.2011.00550.x 22074331 PMC3557465

[38] Wiley CA, Bonneh-Barkay D, Dixon CE, Lesniak A, Wang G, Bissel SJ, Kochanek PM. Role for mammalian chitinase 3-like protein 1 in traumatic brain injury. Neuropathology. 2015; 35:95–106. 10.1111/neup.12158 25377763

[39] Querol-Vilaseca M, Colom-Cadena M, Pegueroles J, San Martín-Paniello C, Clarimon J, Belbin O, Fortea J, Lleó A. YKL-40 (Chitinase 3-like I) is expressed in a subset of astrocytes in Alzheimer’s disease and other tauopathies. J Neuroinflammation. 2017; 14:118. 10.1186/s12974-017-0893-7 28599675 PMC5466718

[40] Ferrer I. Diversity of astroglial responses across human neurodegenerative disorders and brain aging. Brain Pathol. 2017; 27:645–74. 10.1111/bpa.12538 28804999 PMC8029391

[41] Pagliardini V, Pagliardini S, Corrado L, Lucenti A, Panigati L, Bersano E, Servo S, Cantello R, D’Alfonso S, Mazzini L. Chitotriosidase and lysosomal enzymes as potential biomarkers of disease progression in amyotrophic lateral sclerosis: a survey clinic-based study. J Neurol Sci. 2015; 348:245–50. 10.1016/j.jns.2014.12.016 25563799

[42] Steinacker P, Verde F, Fang L, Feneberg E, Oeckl P, Roeber S, Anderl-Straub S, Danek A, Diehl-Schmid J, Fassbender K, Fliessbach K, Foerstl H, Giese A, et al, and FTLDc study group. Chitotriosidase (CHIT1) is increased in microglia and macrophages in spinal cord of amyotrophic lateral sclerosis and cerebrospinal fluid levels correlate with disease severity and progression. J Neurol Neurosurg Psychiatry. 2018; 89:239–47. 10.1136/jnnp-2017-317138 29142138

[43] Sanfilippo C, Longo A, Lazzara F, Cambria D, Distefano G, Palumbo M, Cantarella A, Malaguarnera L, Di Rosa M. CHI3L1 and CHI3L2 overexpression in motor cortex and spinal cord of sALS patients. Mol Cell Neurosci. 2017; 85:162–69. 10.1016/j.mcn.2017.10.001 28989002

[44] Thompson AG, Gray E, Thézénas ML, Charles PD, Evetts S, Hu MT, Talbot K, Fischer R, Kessler BM, Turner MR. Cerebrospinal fluid macrophage biomarkers in amyotrophic lateral sclerosis. Ann Neurol. 2018; 83:258–68. 10.1002/ana.25143 29331073

[45] Illán-Gala I, Alcolea D, Montal V, Dols O, Muñoz L, de Luna N, Turón-Sans J, Cortés-Vicente E, Sánchez-Saudinós B, Subirana A, Sala I, Blesa R, Clarimón J, et al. CSF sAPPβ, YKL-40, and NfL along the ALS-FTD spectrum. Neurology. 2017; 89:178–88. 10.1212/WNL.0000000000004088. 30291183

[46] Fujita K, Kato T, Yamauchi M, Ando M, Honda M, Nagata Y. Increases in fragmented glial fibrillary acidic protein levels in the spinal cords of patients with amyotrophic lateral sclerosis. Neurochem Res. 1998; 23:169–74. 10.1023/A:1022476724381 9475511

[47] Graves MC, Fiala M, Dinglasan LA, Liu NQ, Sayre J, Chiappelli F, van Kooten C, Vinters HV. Inflammation in amyotrophic lateral sclerosis spinal cord and brain is mediated by activated macrophages, mast cells and T cells. Amyotroph Lateral Scler Other Motor Neuron Disord. 2004; 5:213–19. 10.1080/14660820410020286 15799549

[48] Henkel JS, Engelhardt JI, Siklós L, Simpson EP, Kim SH, Pan T, Goodman JC, Siddique T, Beers DR, Appel SH. Presence of dendritic cells, MCP-1, and activated microglia/macrophages in amyotrophic lateral sclerosis spinal cord tissue. Ann Neurol. 2004; 55:221–35. 10.1002/ana.10805 14755726

[49] Calvo A, Moglia C, Balma M, Chiò A. Involvement of immune response in the pathogenesis of amyotrophic lateral sclerosis: a therapeutic opportunity? CNS Neurol Disord Drug Targets. 2010; 9:325–30. 10.2174/187152710791292657 20406178

[50] Casula M, Iyer AM, Spliet WG, Anink JJ, Steentjes K, Sta M, Troost D, Aronica E. Toll-like receptor signaling in amyotrophic lateral sclerosis spinal cord tissue. Neuroscience. 2011; 179:233–43. 10.1016/j.neuroscience.2011.02.001 21303685

[51] Sta M, Sylva-Steenland RM, Casula M, de Jong JM, Troost D, Aronica E, Baas F. Innate and adaptive immunity in amyotrophic lateral sclerosis: evidence of complement activation. Neurobiol Dis. 2011; 42:211–20. 10.1016/j.nbd.2011.01.002 21220013

[52] McCombe PA, Henderson RD. The Role of immune and inflammatory mechanisms in ALS. Curr Mol Med. 2011; 11:246–54. 10.2174/156652411795243450 21375489 PMC3182412

[53] Philips T, Robberecht W. Neuroinflammation in amyotrophic lateral sclerosis: role of glial activation in motor neuron disease. Lancet Neurol. 2011; 10:253–63. 10.1016/S1474-4422(11)70015-1 21349440

[54] Evans MC, Couch Y, Sibson N, Turner MR. Inflammation and neurovascular changes in amyotrophic lateral sclerosis. Mol Cell Neurosci. 2013; 53:34–41. 10.1016/j.mcn.2012.10.008 23110760

[55] Hooten KG, Beers DR, Zhao W, Appel SH. Protective and toxic neuroinflammation in amyotrophic lateral sclerosis. Neurotherapeutics. 2015; 12:364–75. 10.1007/s13311-014-0329-3 25567201 PMC4404435

[56] Komine O, Yamanaka K. Neuroinflammation in motor neuron disease. Nagoya J Med Sci. 2015; 77:537–49. 26663933 PMC4664586

[57] Puentes F, Malaspina A, van Noort JM, Amor S. Non-neuronal cells in ALS: role of glial, immune cells and blood-CNS barriers. Brain Pathol. 2016; 26:248–57. 10.1111/bpa.12352 26780491 PMC8029111

[58] Andrés-Benito P, Moreno J, Aso E, Povedano M, Ferrer I. Amyotrophic lateral sclerosis, gene deregulation in the anterior horn of the spinal cord and frontal cortex area 8: implications in frontotemporal lobar degeneration. Aging (Albany NY). 2017; 9:823–51. 10.18632/aging.101195 28283675 PMC5391234

[59] Liu J, Wang F. Role of neuroinflammation in amyotrophic lateral sclerosis: cellular mechanisms and therapeutic implications. Front Immunol. 2017; 8:1005. 10.3389/fimmu.2017.01005 28871262 PMC5567007

[60] Sekizawa T, Openshaw H, Ohbo K, Sugamura K, Itoyama Y, Niland JC. Cerebrospinal fluid interleukin 6 in amyotrophic lateral sclerosis: immunological parameter and comparison with inflammatory and non-inflammatory central nervous system diseases. J Neurol Sci. 1998; 154:194–99. 10.1016/S0022-510X(97)00228-1 9562310

[61] Rentzos M, Nikolaou C, Rombos A, Boufidou F, Zoga M, Dimitrakopoulos A, Tsoutsou A, Vassilopoulos D. RANTES levels are elevated in serum and cerebrospinal fluid in patients with amyotrophic lateral sclerosis. Amyotroph Lateral Scler. 2007; 8:283–87. 10.1080/17482960701419232 17852013

[62] Kuhle J, Lindberg RL, Regeniter A, Mehling M, Steck AJ, Kappos L, Czaplinski A. Increased levels of inflammatory chemokines in amyotrophic lateral sclerosis. Eur J Neurol. 2009; 16:771–74. 10.1111/j.1468-1331.2009.02560.x 19236470

[63] Mitchell RM, Freeman WM, Randazzo WT, Stephens HE, Beard JL, Simmons Z, Connor JR. A CSF biomarker panel for identification of patients with amyotrophic lateral sclerosis. Neurology. 2009; 72:14–19. 10.1212/01.wnl.0000333251.36681.a5 18987350

[64] Rentzos M, Rombos A, Nikolaou C, Zoga M, Zouvelou V, Dimitrakopoulos A, Alexakis T, Tsoutsou A, Samakovli A, Michalopoulou M, Evdokimidis I. Interleukin-15 and interleukin-12 are elevated in serum and cerebrospinal fluid of patients with amyotrophic lateral sclerosis. Eur Neurol. 2010; 63:285–90. 10.1159/000287582 20407265

[65] Rentzos M, Rombos A, Nikolaou C, Zoga M, Zouvelou V, Dimitrakopoulos A, Alexakis T, Tsoutsou A, Samakovli A, Michalopoulou M, Evdokimidis J. Interleukin-17 and interleukin-23 are elevated in serum and cerebrospinal fluid of patients with ALS: a reflection of Th17 cells activation? Acta Neurol Scand. 2010; 122:425–29. 10.1111/j.1600-0404.2010.01333.x 20219021

[66] Fiala M, Chattopadhay M, La Cava A, Tse E, Liu G, Lourenco E, Eskin A, Liu PT, Magpantay L, Tse S, Mahanian M, Weitzman R, Tong J, et al. IL-17A is increased in the serum and in spinal cord CD8 and mast cells of ALS patients. J Neuroinflammation. 2010; 7:76. 10.1186/1742-2094-7-76 21062492 PMC2992053

[67] Tateishi T, Yamasaki R, Tanaka M, Matsushita T, Kikuchi H, Isobe N, Ohyagi Y, Kira J. CSF chemokine alterations related to the clinical course of amyotrophic lateral sclerosis. J Neuroimmunol. 2010; 222:76–81. 10.1016/j.jneuroim.2010.03.004 20381883

[68] Italiani P, Carlesi C, Giungato P, Puxeddu I, Borroni B, Bossù P, Migliorini P, Siciliano G, Boraschi D. Evaluating the levels of interleukin-1 family cytokines in sporadic amyotrophic lateral sclerosis. J Neuroinflammation. 2014; 11:94. 10.1186/1742-2094-11-94 24884937 PMC4039322

[69] Martínez HR, Escamilla-Ocañas CE, Camara-Lemarroy CR, González-Garza MT, Moreno-Cuevas J, García Sarreón MA. Increased cerebrospinal fluid levels of cytokines monocyte chemoattractant protein-1 (MCP-1) and macrophage inflammatory protein-1β (MIP-1β) in patients with amyotrophic lateral sclerosis. Neurologia. 2017. Epub ahead of print. 10.1016/j.nrl.2017.07.020 29029824

[70] Moreau C, Gosset P, Brunaud-Danel V, Lassalle P, Degonne B, Destee A, Defebvre L, Devos D. CSF profiles of angiogenic and inflammatory factors depend on the respiratory status of ALS patients. Amyotroph Lateral Scler. 2009; 10:175–81. 10.1080/17482960802651725 19177252

[71] Zhang R, Gascon R, Miller RG, Gelinas DF, Mass J, Hadlock K, Jin X, Reis J, Narvaez A, McGrath MS. Evidence for systemic immune system alterations in sporadic amyotrophic lateral sclerosis (sALS). J Neuroimmunol. 2005; 159:215–24. 10.1016/j.jneuroim.2004.10.009 15652422

[72] Shi N, Kawano Y, Tateishi T, Kikuchi H, Osoegawa M, Ohyagi Y, Kira J. Increased IL-13-producing T cells in ALS: positive correlations with disease severity and progression rate. J Neuroimmunol. 2007; 182:232–35. 10.1016/j.jneuroim.2006.10.001 17097743

[73] Cereda C, Baiocchi C, Bongioanni P, Cova E, Guareschi S, Metelli MR, Rossi B, Sbalsi I, Cuccia MC, Ceroni M. TNF and sTNFR1/2 plasma levels in ALS patients. J Neuroimmunol. 2008; 194:123–31. 10.1016/j.jneuroim.2007.10.028 18083240

[74] Mantovani S, Garbelli S, Pasini A, Alimonti D, Perotti C, Melazzini M, Bendotti C, Mora G. Immune system alterations in sporadic amyotrophic lateral sclerosis patients suggest an ongoing neuroinflammatory process. J Neuroimmunol. 2009; 210:73–79. 10.1016/j.jneuroim.2009.02.012 19307024

[75] Rentzos M, Evangelopoulos E, Sereti E, Zouvelou V, Marmara S, Alexakis T, Evdokimidis I. Alterations of T cell subsets in ALS: a systemic immune activation? Acta Neurol Scand. 2012; 125:260–64. 10.1111/j.1600-0404.2011.01528.x 21651502

[76] Rentzos M, Evangelopoulos E, Sereti E, Zouvelou V, Marmara S, Alexakis T, Evdokimidis I. Alterations of T cell subsets in ALS: a systemic immune activation? Acta Neurol Scand. 2012; 125:260–64. 10.1111/j.1600-0404.2011.01528.x 21651502

[77] Henkel JS, Beers DR, Wen S, Rivera AL, Toennis KM, Appel JE, Zhao W, Moore DH, Powell SZ, Appel SH. Regulatory T-lymphocytes mediate amyotrophic lateral sclerosis progression and survival. EMBO Mol Med. 2013; 5:64–79. 10.1002/emmm.201201544 23143995 PMC3569654

[78] Zhao W, Beers DR, Hooten KG, Sieglaff DH, Zhang A, Kalyana-Sundaram S, Traini CM, Halsey WS, Hughes AM, Sathe GM, Livi GP, Fan GH, Appel SH. Characterization of gene expression phenotype in amyotrophic lateral sclerosis monocytes. JAMA Neurol. 2017; 74:677–85. 10.1001/jamaneurol.2017.0357 28437540 PMC5822209

[79] Sidaway P. Motor neuron disease: peripheral immune cell levels correlate with disease progression in ALS. Nat Rev Neurol. 2017; 13:708. 10.1038/nrneurol.2017.149 29027543

[80] Andrés-Benito P, Moreno J, Domínguez R, Aso E, Povedano M, Ferrer I. Inflammatory Gene expression in whole peripheral blood at early stages of sporadic amyotrophic lateral sclerosis. Front Neurol. 2017; 8:546. 10.3389/fneur.2017.00546 29081763 PMC5645505

[81] Weydt P, Oeckl P, Huss A, Müller K, Volk AE, Kuhle J, Knehr A, Andersen PM, Prudlo J, Steinacker P, Weishaupt JH, Ludolph AC, Otto M. Neurofilament levels as biomarkers in asymptomatic and symptomatic familial amyotrophic lateral sclerosis. Ann Neurol. 2016; 79:152–58. 10.1002/ana.24552 26528863

[82] Steinacker P, Feneberg E, Weishaupt J, Brettschneider J, Tumani H, Andersen PM, von Arnim CA, Böhm S, Kassubek J, Kubisch C, Lulé D, Müller HP, Muche R, et al. Neurofilaments in the diagnosis of motoneuron diseases: a prospective study on 455 patients. J Neurol Neurosurg Psychiatry. 2016; 87:12–20. 10.1136/jnnp-2015-311387 26296871

[83] Oeckl P, Jardel C, Salachas F, Lamari F, Andersen PM, Bowser R, de Carvalho M, Costa J, van Damme P, Gray E, Grosskreutz J, Hernández-Barral M, Herukka SK, et al. Multicenter validation of CSF neurofilaments as diagnostic biomarkers for ALS. Amyotroph Lateral Scler Frontotemporal Degener. 2016; 17:404–13. 10.3109/21678421.2016.1167913 27415180

[84] Feneberg E, Oeckl P, Steinacker P, Verde F, Barro C, Van Damme P, Gray E, Grosskreutz J, Jardel C, Kuhle J, Koerner S, Lamari F, Amador MD, et al. Multicenter evaluation of neurofilaments in early symptom onset amyotrophic lateral sclerosis. Neurology. 2018; 90:e22–30. 10.1212/WNL.0000000000004761 29212830

[85] Xu Z, Henderson RD, David M, McCombe PA. Neurofilaments as biomarkers for amyotrophic lateral sclerosis: A systematic review and meta-analysis. PLoS One. 2016; 11:e0164625. 10.1371/journal.pone.0164625 27732645 PMC5061412

[86] Rossi D, Volanti P, Brambilla L, Colletti T, Spataro R, La Bella V. CSF neurofilament proteins as diagnostic and prognostic biomarkers for amyotrophic lateral sclerosis. J Neurol. 2018; 265:510–21. 10.1007/s00415-017-8730-6 29322259

[87] Ferrer I. Brain Banking. In: Aminoff MJ, Daroff RB (eds.) Encyclopedia of the Neurological Sciences, 2nd edition. Oxford: Academic Press 2014; 1, pp. 467-473.

[88] Ince PG, Highley JR, Wharton SB. Motor neuron disorders. In: Love S, Budka H, Ironside JW, Perry A (eds.). Greenfield’s Neuropathology, Ninth edition. Boca Raton: CRC Press, Taylor and Francis Group. 2015, pp. 817-848.

[89] Strong MJ, Hortobágyi T, Okamoto K, Kato S. Amyotrophic lateral sclerosis, primary lateral sclerosis, and spinal muscular atrophy. In: Dickson DW, Weller R.O (eds.) Neurodegeneration: the molecular pathology of dementia and movement disorders. 2^nd^ edition. Oxford: Wiley-Blackwell. 2011, pp. 418-433.

[90] Barrachina M, Castaño E, Ferrer I. TaqMan PCR assay in the control of RNA normalization in human post-mortem brain tissue. Neurochem Int. 2006; 49:276–84. 10.1016/j.neuint.2006.01.018 16522342

[91] Durrenberger PF, Fernando FS, Magliozzi R, Kashefi SN, Bonnert TP, Ferrer I, Seilhean D, Nait-Oumesmar B, Schmitt A, Gebicke-Haerter PJ, Falkai P, Grünblatt E, Palkovits M, et al. Selection of novel reference genes for use in the human central nervous system: a BrainNet Europe Study. Acta Neuropathol. 2012; 124:893–903. 10.1007/s00401-012-1027-z 22864814

[92] Zampieri M, Ciccarone F, Guastafierro T, Bacalini MG, Calabrese R, Moreno-Villanueva M, Reale A, Chevanne M, Bürkle A, Caiafa P. Validation of suitable internal control genes for expression studies in aging. Mech Ageing Dev. 2010; 131:89–95. 10.1016/j.mad.2009.12.005 20038437

[93] Bayatti N, Cooper-Knock J, Bury JJ, Wyles M, Heath PR, Kirby J, Shaw PJ. Comparison of blood RNA extraction methods used for gene expression profiling in amyotrophic lateral sclerosis. PLoS One. 2014; 9:e87508. 10.1371/journal.pone.0087508 24475299 PMC3903649

[94] Youden WJ. Index for rating diagnostic tests. Cancer. 1950; 3:32–35. 10.1002/1097-0142(1950)3:1<32::AID-CNCR2820030106>3.0.CO;2-3 15405679

