# Correction for: YKL40 in sporadic amyotrophic lateral sclerosis: cerebrospinal fluid levels as a prognosis marker of disease progression

**DOI:** 10.18632/aging.203667

**Published:** 2021-10-31

**Authors:** Pol Andrés‐Benito, Raúl Domínguez, Maria J. Colomina, Franc Llorens, Mònica Povedano, Isidre Ferrer

**Affiliations:** 1 Department of Pathology and Experimental Therapeutics, University of Barcelona, L’Hospitalet de Llobregat, Barcelona, Spain; 2 Biomedical Network Research Center on Neurodegenerative Diseases (CIBERNED), Institute Carlos III, L’Hospitalet de Llobregat, Barcelona, Spain; 3 Bellvitge Biomedical Research Institute (IDIBELL), L’Hospitalet de Llobregat, Barcelona, Spain; 4 Functional Unit of Amyotrophic Lateral Sclerosis (UFELA), Service of Neurology, Bellvitge University Hospital, L’Hospitalet de Llobregat, Barcelona, Spain; 5 Anesthesia and Critical Care Department, Bellvitge University Hospital ‐ University of Barcelona L’Hospitalet de Llobregat, Barcelona, Spain; 6 Neuropathology, Pathologic Anatomy Service, Bellvitge University Hospital, IDIBELL, L’Hospitalet de Llobregat, Barcelona, Spain; 7 Institute of Neurosciences, University of Barcelona, Barcelona, Spain

**Keywords:** correction

Original article: Aging. 2018; 10:2367–2382.  . https://doi.org/10.18632/aging.101551

**This article has been corrected:** YKL40 values in text and figures represent **ng/mL**, not pg/mL This alteration does not affect the results or conclusions of this work.

